# Cerebral hemorrhage associated with snakebite by *Bothrops* spp. juvenile: experience of two cases in an Amazonian referral hospital

**DOI:** 10.17843/rpmesp.2026.433.15938

**Published:** 2026-09-28

**Authors:** Edgar A. Ramírez-García, Jorge Sibina Vela, Alfrando Morero, Marco Paredes-Obando, Lidia P. Noriega-Trigoso, Jhosephi J. Vasquez-Ascate, David Crispin-Alvear, Guillermo Velazco-Curay, Juan C. Celis-Salinas, Martín Casapia Morales

**Affiliations:** 1 Universidad Nacional de la Amazonía Peruana, Iquitos, Peru. Universidad Nacional de la Amazonía Peruana Iquitos Peru; 2 Departamento de Enfermedades infecciosas y Tropicales, Hospital Regional de Loreto, Iquitos, Peru. Departamento de Enfermedades infecciosas y Tropicales Hospital Regional de Loreto Iquitos Peru; 3 Instituto Nacional de Ciencias Neurológicas, Lima, Peru. Instituto Nacional de Ciencias Neurológicas Lima Peru

**Keywords:** Bothrops, Snakebites, Intracranial Hemorrhages, Stroke

## Abstract

Snakes of the genus *Bothrops* cause most snakebites in Peru. Among its complications, intracranial hemorrhage is infrequent, but potentially lethal. We describe two cases of hemoventricle after bites by juvenile specimens (<50 cm) in Loreto. The first case corresponds to a 58-year-old male with untreated arterial hypertension, who received antivenom in fractionated doses due to shortage. On the fourth day, he presented sudden headache and loss of consciousness; tomography revealed hemorrhage in the fourth ventricle. The second case corresponds to a five-year-old child who received traditional medicine and whose first dose of antivenom was administered after four days of the bite, following a prolonged fluvial transfer. He presented severe anemia, altered sensorium, and hemorrhage in the left lateral ventricle. Both patients required intensive care, additional antivenom, and supportive measures, and were discharged without neurological deficit. These cases illustrate that delayed or insufficient access to antivenom in the Amazon can be associated with hemorrhagic complications of the central nervous system.

## INTRODUCTION

*Bothrops* spp. causes the largest proportion of snakebites in South America, especially in the Amazon. Its envenomation can cause hemorrhagic manifestations and severe coagulopathies ^1^^,^^2^. Neurological complications, such as intracranial hemorrhages, are rare but highly lethal. In a series of 309 patients with *Bothrops* bites in Ecuador, 2.6% presented cerebrovascular events, mainly intracranial hemorrhages with an unfavorable prognosis (high mortality or irreversible sequelae) ^3^. In Peru, snake envenomation constitutes the leading cause of fatal envenomations produced by venomous animals each year. Between 2020 and 2021, 2382 cases of snake envenomation have been reported, with Loreto being the most affected region ^4^.

The probable mechanism involves the action of hemorrhagic venom components, such as metalloproteinases and serine proteinases, which injure the vascular endothelium and alter coagulation, promoting hemorrhages in distant sites such as the central nervous system ^5^^,^^6^. The delay in antivenom administration, frequent in rural areas with difficult access, aggravates the risk and severity of these complications ^7^. Therefore, it is relevant to describe two new Peruvian cases of intraventricular hemorrhage after *Bothrops* envenomation, as a contribution to enrich the regional and international literature, and that at the same time highlights the diagnostic and therapeutic challenges in remote contexts.

## CASE REPORT

### Case 1

A 58-year-old male farmer from Requena, with a history of untreated arterial hypertension. The river transfer from this town to Iquitos can take between 10 and 15 hours by motorized boat. While working barefoot, he suffered a bite on the second toe of his left foot by a juvenile *Bothrops* specimen approximately 30 cm long. Initially, he received two vials of antivenom; in addition, local cleaning with alcohol was performed, and tourniquets were applied to the leg. Eight hours later, two additional vials were administered.

In the following days, he presented intense burning pain, progressive edema, local necrosis, and multiple bleeding episodes (gums, hematic sputum, epistaxis, cutaneous hematomas). Due to the lack of antivenom at the Health Center, he was transferred by river and land to Iquitos Hospital, where he received 10 vials of antivenom (figure 1).

**Figure 1:**
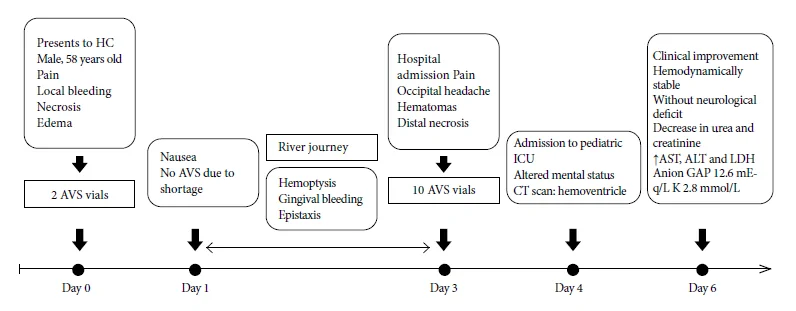
Timeline of Case 1.

HC: health center, AVS: antivenom serum, CT: computed axial tomography, ICU: intensive care unit.

On the fourth day, he presented with sudden headache, visual disturbances, generalized weakness, and loss of consciousness. Initial examinations showed a systemic inflammatory response with leukocytosis, neutrophilia, and moderate anemia, probably secondary to the local hemorrhagic effect of the venom. The biochemical profile showed a progressive elevation of transaminases and an elevated LDH (lactate dehydrogenase), confirming tissue or muscle damage, while the coagulation profile showed a slightly prolonged prothrombin time with fibrinogen and platelets within normal ranges, suggesting controlled factor consumption. However, the most critical finding is progressive moderate to severe hypokalemia, which, along with the marked decrease in urea and the fluctuation of the anion GAP, indicates a metabolic hydroelectrolytic imbalance (table 1). Non-contrast brain computed tomography showed a hyperdense image in the fourth ventricle compatible with hemoventricle (figure 2A), entering the intensive care unit (ICU) with sedation and vasoactive support.

**Table 1:** Biochemical, hematological, blood gas, and electrolyte results of case 1.

Results		Day 3	Day 4	Day 6
Biochemist				
	Urea	NP	24 mg/dl	4 mg/dl
	Creatinine	NP	0.93 mg/dl	0.54 mg/dl
	AST	NP	16 U/l	52 U/l
	ALT	NP	33 U/l	75 U/l
	LDH	NP	713 U/l	549 U/l
	PT	NP	16.4 sec	16.4 sec
	aPTT	NP	42 sec	NP
	Fibrinogen	NP	256.5 mg/dl	NP
Complete Blood Count				
	Leukocytes	NP	10 660 / uL	NP
	Neutrophils	NP	83.1%	NP
	Lymphocytes	NP	9.8%	NP
	Hemoglobin	NP	9.5 g/dL	NP
	Hematocrit	NP	29.3%	NP
	Platelets	NP	310 000 / uL	NP
	Coagulation Time	NP	4 min and 30 sec	NP
	Bleeding time	NP	2 min and 30 sec	NP
Blood Gases and Electrolytes				
	pH	7.441	7.445	7.429
	PCO2	31.2 mmHg	29,2 mmHg	32.8 mmHg
	FiO2	21%	21%	21%
	PO2	88 mmHg	85 mmHg	79 mmHg
	HCO3	21.2 mmol/L	20.1 mmol/L	21.7 mmol/L
	O2 Sat	96.1%	97 %	96.1%
	Na	135 mmol/L	134.2 mmol/L	134.9 mmol/L
	K	3.45 mmol/L	3.05 mmol/L	2.8 mmol/L
	Cl	101.8 mmol/L	98 mmol/L	102.4 mmol/L
	BE ecf	-3.1 mmol/L	-4.2 mmol/L	-2.8 mmol/L
	Anion GAP	14.4 mEq/L	18.3 mEq/L	12.6 mEq/L

NP: not performed

**Figure 2:**
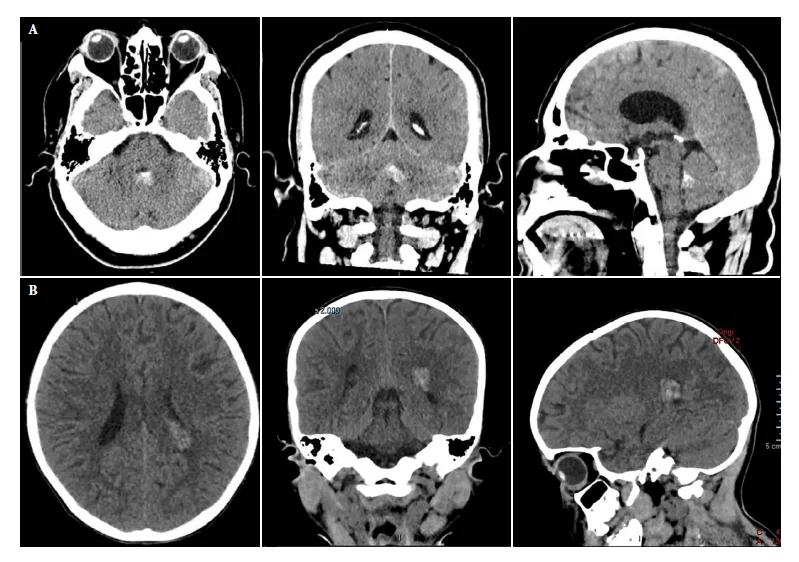
A) Case 1. Non-contrast brain computed tomography showing a hyperdense image located in the fourth ventricle compatible with hemoventricle. B) Case 2. Non-contrast brain computed tomography in axial, coronal, and sagittal views. A hyperdense image is observed located in the occipital horn of the left lateral ventricle compatible with a left unilateral hemoventricle.

On the sixth day, the patient was hemodynamically stable and without neurological deficit. Urea decreased to 4 mg/dL and creatinine to 0.54 mg/dL. Transaminases increased (AST 52 U/L and ALT 75 U/L), while LDH decreased to 549 U/L; potassium was 2.8 mmol/L (table 1).

After five days of specialized management in the ICU and with the stabilization of the systemic inflammatory response, the patient was transferred to the conventional hospitalization area. The restoration of metabolic and coagulation values was achieved, as well as the resolution of initial anemia and leukocytosis. The patient was discharged with comprehensive clinical improvement and without detectable neurological sequelae, demonstrating a satisfactory recovery after a case of ophidism with central nervous system involvement.

### Case 2

Five-year-old male, with no relevant medical history, from the Palometa hamlet (Napo River, Loreto), approximately 965 km from Iquitos and a 12-hour river trip away. Five days earlier, while playing barefoot, he suffered a bite on the dorsomedial region of the left first toe by a juvenile *Bothrops* specimen approximately 20 cm long (supplementary material). Initially, he presented with pain and local bleeding, followed by progressive edema of the foot and leg, distal necrosis, and spontaneous gingival bleeding. He received oral ampicillin and subsequently, traditional medicine.

On the third day, he presented fever, persistent postprandial vomiting, marked pallor, and generalized ecchymoses. On the fourth day, his transfer began, and after 15 hours of travel, he arrived at the Libertad health post, where he only received hydration. Subsequently, he was transferred to the I-3 Mazán Health Center, where five vials of antivenom were administered to him, and finally to the Regional Hospital of Loreto (figure 3).

**Figure 3:**
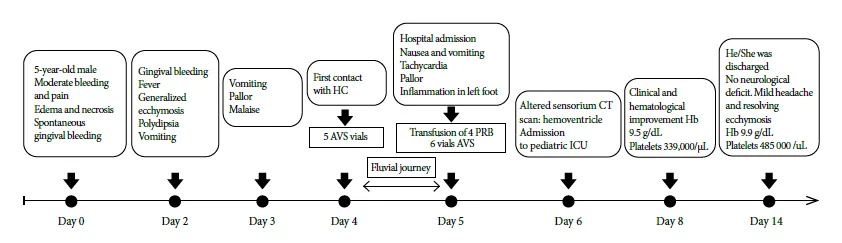
Timeline of Case 2.

HC: health center. PRB: packed red blood cells, AVS: antivenom, CT: computed tomography scan, ICU: intensive care unit.

Upon admission, the patient presented with severe pallor, hypotension (BP: 90/50 mmHg), tachycardia (135 bpm), tachypnea (36 rpm), persistent vomiting, inflammation in the left foot, and severe anemia. The patient received intravenous fluids, antibiotics, analgesia, transfusion of four units of packed red blood cells, and six vials of antivenom, with a subsequent additional transfusion.

During hospitalization, he developed altered sensorium and nuchal rigidity; brain tomography revealed hemoventricle in the occipital horn of the left lateral ventricle (figure 2B). He was admitted to the pediatric ICU, where he received oxygen via nasal cannula and supportive treatment. After an episode of life-threatening severe anemia and thrombocytopenia on days 5 and 6, clinical support allowed for robust hematological recovery by day 14, with stable hemoglobin and reactive thrombocytosis. Concurrently, the significant uremia resolved (urea decreased from 112.1 to 30 mg/dL) and hemostasis normalized. Despite residual tissue damage evidenced by elevated LDH (812.24 U/L), the appearance of marked eosinophilia (18.4%) at the end of the period suggests a late immunological reaction, possibly associated with the administration of antivenom or the resolution phase of the systemic inflammatory process (table 2).

**Table 2:** Evolution of hematological results of case 2.

Complete Blood Count	Day 5	Day 6	Day 8	Day 14
Leukocytes	11 330 / uL	14 640 / uL	16 550 / uL	12 920 / uL
Neutrophils	70.1%	80.6%	77.6%	57.1%
Lymphocytes	19.9%	11.6%	14%	16.5%
Monocytes	9.5%	6.5%	5.0%	7.5%
Eosinophils	0.1%	0.9%	3.0%	18.4%
Basophils	0.4%	0.4%	0.4%	0.5%
Hb	6.2 g/dL	10.3 g/dL	9.5 g/dL	9.9 g/dL
Hct	19.3%	32%	29.2%	30.5%
MCV	88.7 fL	90.8 fL	90.1 fL	90.6 fL
Platelets	169 000 / uL	137 000 / uL	339 000 / uL	485 000 / uL

Hb: hemoglobin, Hct: hematocrit, MCV: mean corpuscular volume.

After admission on day 5 and a critical period in the pediatric ICU (day 6). On day 8, hematological recovery was observed, with hemoglobin of 9.5 g/dL and platelets of 339,000/µL. The patient presented satisfactory clinical and laboratory recovery. On day 14, he was discharged without neurological deficit, with mild headache and ecchymosis in resolution; hemoglobin was 9.9 g/dL and platelets were 485,000/µL (table 2). The patient was discharged from the hospital after nine days of specialized surveillance, without neurological deficit or meningeal signs, with only mild headache and ecchymosis persisting in the resolution phase.

## DISCUSSION

Snakebites have been classified by the World Health Organization as neglected diseases. In Latin America, most snakebites are caused by species of the Viperidae family, particularly by the genus *Bothrops*^2^. *Bothrops atrox* snakes are found mainly in the Amazon rainforest, although they have also been observed in the Bolivian Yungas, the Colombian plains, and possibly in some humid forest areas of Caatinga, known as «Brejos de Altitude» in northeast Brazil ^3^. Approximately, five million people are bitten by snakes each year, resulting in more than two million cases of envenoming and between 20,000 to 125,000 deaths ^4^.

*Bothrops* venom has proteolytic, coagulant, and hemorrhagic actions ^5^. Envenomation by *Bothrops atrox* snakes produces local and systemic effects, with edema being an important clinical manifestation that can progress to compartment syndromes and in severe cases, cause tissue loss and require amputations. The venom of these snakes activates multiple blood systems related to thromboinflammation, which can influence the pathophysiology of envenomations ^6^.

Local manifestations primarily include pain, edema, and erythema, while systemic ones may include bleeding ^7^. The bite wound can frequently become infected, especially in patients who have suffered grade 2 or 3 envenomation, and in those presenting with necrosis, thrombocytopenia, or rhabdomyolysis. The bacteria most commonly associated with these infections are *Aeromonas hydrophila* and *Morganella morganii*^8^.

Hemorrhagic syndrome remains the most important cause of lethality associated with these species. Other complications include acute kidney injury, shock, myonecrosis, compartment syndrome, and secondary infections ^9^. Hemorrhagic stroke is a complication that can be very frequent in patients with preexisting risk factors, such as diabetes, nephropathies, and cardiovascular diseases ^10^. Coagulant activity is the result of venom components with thrombin-like activity, which directly hydrolyze fibrinogen to fibrin, and procoagulant activity, which activates coagulation factors II and X, resulting in endogenous thrombin formation ^11^. Venom-induced proteolytic activity causes vascular wall lesions that lead to hemorrhages. In hemorrhagic activity, P-I metalloproteinase, especially batroxase, has fibrinolytic and thrombolytic activity and induces bleeding through the digestion of extracellular matrix components ^12^.

The pediatric case corresponds to a five-year-old male who attends the health center due to an ophidian accident by genus *Bothrops*; with five days of evolution. The bite was on the first toe of the left foot. Upon presenting neurological manifestations, a cerebral tomographic study without contrast was requested, where a hyperdense image located in the occipital horn of the left lateral ventricle compatible with unilateral hemoventricle was evidenced.

In a case report in Brazil in 2017, a 58-year-old woman was described who presented with a hemorrhagic stroke two days after suffering a bite from a snake of the genus *Bothrops* on the third finger of the right hand. Cerebral tomography revealed a right nucleocapsular hematoma and hemorrhage in the right ventricular system ^13^.

Also, in a report in Colombia in 2020, a 59-year-old male was described who suffered a bite on the distal part of the right thumb, with 12 hours of evolution. In the plain cranial computed tomography, massive temporoparietal cerebral hemorrhage was observed, compressing the right basal nuclei ^14^.

Hemoventricle is a rare but severe complication of snakebites in children. It occurs when the action of the venom causes hemorrhage in the ventricular system, composed of spaces filled with cerebrospinal fluid. Hemoventricle can cause an increase in pressure within the ventricular system, thereby increasing intracranial pressure and causing severe neurological complications such as hydrocephalus and cerebral herniation syndromes.

The exact mechanisms by which snake venom causes ventricular hemorrhage are not well understood, but are believed to be related to the toxic effects of the venom on blood vessels and the ventricular walls. Children are particularly susceptible to hemoventricle from snakebites because their brain is still developing and may be more sensitive to the toxic effects of the venom. Early recognition and timely therapeutic management of snake envenomation are crucial for preventing serious complications such as hemoventricle. This comprehensive management must include the immediate administration of specific antivenom, along with supportive measures aimed at hemodynamic stabilization, the correction of hydroelectrolytic imbalance, and symptomatic control of pain and the inflammatory process ^15^.

In case of suspected hemoventricle, imaging studies such as computed tomography scans are required to confirm the diagnosis and assess the extent of bleeding. Treatment may include medical management or surgical intervention, depending on the severity of bleeding and the presence of other symptoms. It is important to seek immediate medical attention upon any suspicion of snakebite, especially in children, to minimize the risk of hemoventricle and other potentially life-threatening complications.

Peru has a remarkable biodiversity, and each species presents its own peculiarities. The diversity within the *Bothrops* complex is especially noteworthy, meaning that many native snake species with medical relevance have not yet been sufficiently studied ^16^.

There are few studies or reports that document the epidemiology, pathophysiology, and clinical characteristics of ventricular hemorrhage associated with snakebites. Therefore, it is necessary to conduct studies that allow understanding the pathophysiological mechanisms involved in neurological manifestations and determine why children might present a greater susceptibility. This evidence would contribute to optimizing clinical management and to the development of clinical practice guidelines aimed at preventing complications.

In conclusion, intraventricular hemorrhage is a possible complication of *Bothrops* genus snakebite envenomation, even after bites by juvenile specimens, in both adults and children. In the two described cases, delays in the administration of an adequate dose of antivenom and non-recommended prehospital practices were identified. In the adult, the coexistence of untreated arterial hypertension prevents attributing the hemorrhage exclusively to the venom. Given the appearance of neurological alterations after a bothropic snakebite, urgent neuroimaging is suggested, preferably a non-contrast brain computed tomography, and magnetic resonance imaging should be reserved for selected cases. Ensuring the availability of antivenom in primary care facilities and expediting fluvial transfer to higher-resolution centers in the Amazon could reduce the risk of these complications.
