# Supplementary material for: Cerebral hemorrhage associated with snakebite by Bothrops spp. juvenile: experience of two cases in an Amazonian referral hospital
Source: Rev Peru Med Exp Salud Publica. 2026 Sep 28;43(3):408–14. doi: 10.17843/rpmesp.2026.433.15938 (PMC13718573; doi:10.17843/rpmesp.2026.433.15938)
Supplement: Supplementary file 1 [file rpmesp-43-03-15938-s001-es.docx]

**Material Suplementario**

**Figura 1S.** Puntos de mordedura de aproximadamente 8mm localizados en el dedo gordo del pie izquierdo con ligero edema del pie.
