# Supplementary material for: Cerebral hemorrhage associated with snakebite by Bothrops spp. juvenile: experience of two cases in an Amazonian referral hospital
Source: Rev Peru Med Exp Salud Publica. 2026 Sep 28;43(3):408–14. doi: 10.17843/rpmesp.2026.433.15938 (PMC13718573; doi:10.17843/rpmesp.2026.433.15938)
Supplement: Translation

# Translation: Hemorragia cerebral asociada a accidente ofídico por *Bothrops* spp. juvenil: experiencia de dos casos en un hospital amazónico de referencia

**Authors:** Edgar A. Ramírez-García, Jorge Sibina Vela, Alfrando Morero, Marco Paredes-Obando, Lidia P. Noriega-Trigoso, Jhosephi J. Vasquez-Ascate, David Crispin-Alvear, Guillermo Velazco-Curay, Juan C. Celis-Salinas, Martín Casapia Morales

**Affiliations:** 1 Universidad Nacional de la Amazonía Peruana, Iquitos, Perú.; 2 Departamento de Enfermedades infecciosas y Tropicales, Hospital Regional de Loreto, Iquitos, Perú.; 3 Instituto Nacional de Ciencias Neurológicas, Lima, Perú.

**Keywords:** Bothrops, Mordeduras de Serpientes, Hemorragias Intracraneales, Accidente Cerebrovascular

## Abstract

Las serpientes del género *Bothrops* causan la mayoría de los envenenamientos ofídicos en el Perú. Entre sus complicaciones, la hemorragia intracraneal es infrecuente, pero potencialmente letal. Describimos dos casos de hemoventrículo tras mordeduras por ejemplares juveniles (<50 cm) en Loreto. El primer caso corresponde a un varón de 58 años con hipertensión arterial no tratada, quien recibió antiveneno en dosis fraccionadas debido al desabastecimiento. Al cuarto día presentó cefalea súbita y pérdida de conciencia; la tomografía reveló hemorragia en el cuarto ventrículo. El segundo caso corresponde a un niño de cinco años que recibió medicina tradicional y cuya primera dosis de antiveneno se administró después de cuatro días de la mordedura, tras un traslado fluvial prolongado. Presentó anemia grave, alteración del sensorio y hemorragia en el ventrículo lateral izquierdo. Ambos pacientes requirieron cuidados intensivos, antiveneno adicional y medidas de soporte, y egresaron sin déficit neurológico. Estos casos ilustran que el acceso tardío o insuficiente al antiveneno en la Amazonía puede asociarse con complicaciones hemorrágicas del sistema nervioso central.

## INTRODUCCIÓN

*Bothrops* spp. causa la mayor proporción de las mordeduras de serpientes en América del Sur, especialmente en la Amazonía. Su envenenamiento puede provocar manifestaciones hemorrágicas y coagulopatías graves ^1^^,^^2^. Las complicaciones neurológicas, como hemorragias intracraneales, son poco frecuentes, pero altamente letales. En una serie de 309 pacientes con mordeduras por *Bothrops* en Ecuador, el 2,6% presentó eventos cerebrovasculares, principalmente hemorragias intracraneales con pronóstico desfavorable (mortalidad elevada o secuelas irreversibles) ^3^. En el Perú, el ofidismo constituye la primera causa de envenenamientos fatales producidos por animales ponzoñosos cada año. Entre el 2020 y 2021 se han reportado 2382 casos de ofidismo, siendo Loreto la región más afectada ^4^.

El mecanismo probable involucra la acción de componentes del veneno hemorrágico, como metaloproteinasas y serinproteinasas, que lesionan el endotelio vascular y alteran la coagulación, favoreciendo hemorragias en sitios distantes como el sistema nervioso central ^5^^,^^6^. La demora en la administración de antiveneno, frecuente en zonas rurales de difícil acceso, agrava el riesgo y la gravedad de estas complicaciones ^7^. Por lo tanto, resulta relevante describir dos nuevos casos peruanos de hemorragia intraventricular tras envenenamiento por *Bothrops*, como aporte que enriquezca la literatura regional e internacional, y que al mismo tiempo evidencie los desafíos diagnósticos y terapéuticos en contextos remotos.

## REPORTE DE CASO

### Caso 1

Varón de 58 años, agricultor procedente de Requena, con antecedente de hipertensión arterial no tratada. El traslado fluvial desde esta localidad hasta Iquitos puede tardar entre 10 y 15 horas en deslizador motorizado. Mientras trabajaba descalzo, sufrió una mordedura en el segundo dedo del pie izquierdo por un ejemplar juvenil de Bothrops de aproximadamente 30 cm de longitud. Inicialmente, recibió dos viales de suero antiofídico; además se realizó limpieza local con alcohol y se aplicaron torniquetes en la pierna. Ocho horas después se le administraron dos viales adicionales.

En los días siguientes presentó dolor urente intenso, edema progresivo, necrosis local y múltiples episodios de sangrado (encías, escupitajos hemáticos, epistaxis, hematomas cutáneos). Debido a la falta de suero antiofídico en el centro de salud, fue trasladado por vía fluvial y terrestre al Hospital de Iquitos, donde recibió 10 viales de suero antiofídico (figura 1).

**Figura 1:** Línea de tiempo del caso 1.

CS: centro de salud, SAF: suero antiofídico, TAC: tomografía axial computarizada, UCI: unidad de cuidados intensivos.

Al cuarto día presentó cefalea brusca, alteraciones visuales, debilidad generalizada y pérdida de conciencia. Los exámenes iniciales mostraron respuesta inflamatoria sistémica con leucocitosis, neutrofilia y una anemia moderada probablemente secundaria al efecto hemorrágico local del veneno. En el perfil bioquímico destaca una elevación progresiva de transaminasas y una LDH (lactato deshidrogenasa) elevada que confirman daño tisular o muscular, mientras que el perfil de coagulación muestra un tiempo de protrombina levemente prolongado con fibrinógeno y plaquetas dentro de rangos normales, sugiriendo un consumo de factores controlado. Sin embargo, el hallazgo más crítico es la hipopotasemia moderada a severa progresiva, que junto a la marcada disminución de la urea y la fluctuación del anión GAP, que indica un desequilibrio hidroelectrolítico metabólico (tabla 1). La tomografía cerebral sin contraste mostró imagen hiperdensa en cuarto ventrículo compatible con hemoventrículo (figura 2A), ingresando a la unidad de cuidados intensivos (UCI) con sedación y soporte vasoactivo.

**Tabla 1:** Resultados bioquímicos, hematológicos, gasometría y electrolitos del caso 1.

Resultados		Día 3	Día 4	Día 6
Bioquímico				
	Urea	NR	24 mg/dl	4 mg/dl
	Creatinina	NR	0.93 mg/dl	0.54 mg/dl
	TGO	NR	16 U/l	52 U/l
	TGP	NR	33 U/l	75 U/l
	LDH	NR	713 U/l	549 U/l
	TP	NR	16,4 seg	16,4 seg
	TTPA	NR	42 seg	NR
	Fibrinógeno	NR	256,5 mg/dl	NR
Hemograma				
	Leucocitos	NR	10 660 / uL	NR
	Neutrófilos	NR	83,1%	NR
	Linfocitos	NR	9,8%	NR
	Hemoglobina	NR	9,5 g/dL	NR
	Hematocrito	NR	29,3%	NR
	Plaquetas	NR	310 000 / uL	NR
	Tiempo Coagulación	NR	4 min y 30 seg	NR
	Tiempo de sangría	NR	2 min y 30 seg	NR
Gasometría y electrolitos				
	pH	7,441	7,445	7,429
	PCO2	31,2 mmHg	29,2 mmHg	32,8 mmHg
	FiO2	21%	21%	21%
	PO2	88 mmHg	85 mmHg	79 mmHg
	HCO3	21,2 mmol/L	20,1 mmol/L	21,7 mmol/L
	Sat O2	96,1%	97 %	96,1%
	Na	135 mmol/L	134,2 mmol/L	134,9 mmol/L
	K	3,45 mmol/L	3,05 mmol/L	2,8 mmol/L
	Cl	101,8 mmol/L	98 mmol/L	102,4 mmol/L
	BE ecf	-3,1 mmol/L	-4,2 mmol/L	-2,8 mmol/L
	Anión GAP	14,4 mEq/L	18,3 mEq/L	12,6 mEq/L

NR: no realizado

**Figura 2:** A) Caso 1. Tomografía cerebral computarizada sin contraste que impresiona una imagen hiperdensa localizada en el cuarto ventrículo compatible con hemoventrículo. B) Caso 2. Tomografía cerebral computarizada sin contraste en cortes axial, coronal y sagital. Se observa imagen hiperdensa localizada en la asta occipital del ventrículo lateral izquierdo compatible con un hemoventrículo unilateral izquierdo.

Al sexto día, el paciente se encontraba hemodinámicamente estable y sin déficit neurológico. La urea descendió a 4 mg/dL y la creatinina a 0,54 mg/dL. Las transaminasas se elevaron (TGO 52 U/L y TGP 75 U/L), mientras que la LDH disminuyó a 549 U/L; el potasio fue de 2,8 mmol/L (tabla 1).

Tras cinco días de manejo especializado en la UCI y con la estabilización de la respuesta inflamatoria sistémica, el paciente fue trasladado al área de hospitalización convencional. Se logró el restablecimiento de los valores metabólicos y de coagulación, así como la resolución de la anemia y la leucocitosis inicial. El paciente fue dado de alta con una mejoría clínica integral y sin secuelas neurológicas detectables, demostrando una recuperación satisfactoria tras un cuadro de ofidismo con compromiso del sistema nervioso central.

### Caso 2

Varón de cinco años, sin antecedentes relevantes, procedente del caserío Palometa (río Napo, Loreto), a aproximadamente 965 km de Iquitos y a 12 horas de viaje fluvial. Cinco días antes, mientras jugaba descalzo, sufrió una mordedura en la región dorso-medial del primer dedo del pie izquierdo por un ejemplar juvenil de *Bothrops* de aproximadamente de 20 cm de longitud (material suplementario). Inicialmente, presentó dolor y sangrado local, seguido de edema progresivo del pie y la pierna, necrosis distal y gingivorragia espontánea. Recibió ampicilina por vía oral y posteriormente, medicina tradicional.

En el tercer día presentó fiebre, vómitos postprandiales persistentes, palidez marcada y equimosis generalizadas. Al cuarto día se inició su traslado, y tras 15 horas de viaje, llego a la posta de Libertad, donde recibió únicamente hidratación. Posteriormente, fue trasladado al Centro de Salud I-3 Mazán, donde le administraron cinco viales de suero antiofídico, y finalmente al Hospital Regional de Loreto (figura 3).

**Figura 3:** Línea de tiempo del caso 2

CS: centro de salud. PG: paquete globular, SAF: suero antiofídico, TAC: tomografía axial computarizada, UCI: unidad de cuidados intensivos.

Al ingreso, presentó palidez intensa, hipotensión (PA: 90/50 mmHg), taquicardia (135 lpm), taquipnea (36 rpm), vómitos persistentes, flogosis en pie izquierdo y anemia severa. Recibio líquidos intravenosos, antibióticos, analgesia, transfusión de cuatro paquetes globulares y seis viales de suero antiofídico, con transfusión adicional posterior.

Durante la hospitalización desarrolló alteración del sensorio y rigidez de nuca; la tomografía cerebral evidenció hemoventrículo en asta occipital del ventrículo lateral izquierdo (figura 2B). Ingresó a la UCI pediátrica, donde recibio oxígeno por cánula nasal y tratamiento de soporte. Tras un episodio de anemia severa de riesgo vital y trombocitopenia en los días 5 y 6, el soporte clínico permitió una recuperación hematológica robusta hacia el día 14, con una hemoglobina estable y una trombocitosis reactiva. Paralelamente, se resolvió el cuadro de uremia importante (descenso de urea de 112,1 a 30 mg/dL) y se normalizó la hemostasia. A pesar del daño tisular residual evidenciado por la LDH elevada (812.24 U/l), la aparición de una eosinofilia marcada (18,4%) al final del periodo sugiere una reacción inmunológica tardía, posiblemente asociada a la administración del suero antiofídico o a la fase de resolución del proceso inflamatorio sistémico (tabla 2).

**Tabla 2:** Evolución de los resultados hematológicos del caso 2.

Hemograma	Día 5	Día 6	Día 8	Día 14
Leucocitos	11 330 / uL	14 640 / uL	16 550 / uL	12 920 / uL
Neutrófilos	70,1%	80,6%	77,6%	57,1%
Linfocitos	19,9%	11,6%	14%	16,5%
Monocitos	9,5%	6,5%	5,0%	7,5%
Eosinófilos	0,1%	0,9%	3,0%	18,4%
Basófilos	0,4%	0,4%	0,4%	0,5%
Hb	6,2 g/dL	10,3 g/dL	9,5 g/dL	9,9 g/dL
Hct	19,3%	32%	29,2%	30,5%
VCM	88,7 fL	90,8 fL	90,1 fL	90,6 fL
Plaquetas	169 000 / uL	137 000 / uL	339 000 / uL	485 000 / uL

Hb: hemoglobina, Hct: hematocrito: VCM: volumen corpuscular medio.

Tras su ingreso el día 5 y un periodo crítico en la UCI pediátrica (día 6). Al día 8 se observó recuperación hematológica, con hemoglobina de 9,5 g/dL y plaquetas de 339 000/µL. El paciente presentó una recuperación clínica y laboratorial satisfactoria. Al día 14 recibió el alta sin déficit neurológico, con cefalea leve y equimosis en resolución; la hemoglobina fue de 9,9 g/dL y las plaquetas de 485 000/µL (tabla 2). El paciente se retiró del hospital tras nueve días de vigilancia especializada, sin déficit neurológico ni signos meníngeos, persistiendo únicamente cefalea leve y equimosis en fase de resolución.

## DISCUSIÓN

Las mordeduras por serpientes han sido clasificadas por la Organización Mundial de la Salud como enfermedades desatendidas. En América Latina, la mayoría de las mordeduras de serpientes son causadas por especies de la familia Viperidae, en particular por el género *Bothrops*^2^. Las serpientes *Bothrops atrox* se encuentran principalmente en la selva amazónica, aunque también se han observado en las Yungas bolivianas, los llanos colombianos y posiblemente en algunas áreas de bosques húmedos de Caatinga, conocidas como «Brejos de Altitude» en el noreste de Brasil ^3^. Aproximadamente, cinco millones de personas son mordidas por serpientes cada año, lo que resulta en más de dos millones de casos de envenenamiento y entre 20 000 a 125 000 muertes ^4^.

El veneno de *Bothrops* tiene acciones proteolíticas, coagulantes y hemorrágicas ^5^. El envenenamiento por serpientes *Bothrops atrox* produce efectos locales y sistémicos, siendo el edema una manifestación clínica importante que puede progresar a síndromes compartimentales y en casos graves, ocasionar pérdida de tejido y requerir amputaciones. El veneno de estas serpientes activa múltiples sistemas sanguíneos relacionados con la tromboinflamación, lo que puede influir en la fisiopatología de los envenenamientos ^6^.

Las manifestaciones locales incluyen principalmente dolor, edema y eritema, mientras que las sistémicas pueden incluir sangrado ^7^. La herida de la mordedura puede infectarse con frecuencia, especialmente en pacientes que han sufrido un envenenamiento de grado 2 o 3, y en quienes presentan necrosis, trombocitopenia o rabdomiólisis. Las bacterias más comúnmente asociadas con estas infecciones son *Aeromonas hydrophila* y *Morganella morganii*^8^.

El síndrome hemorrágico sigue siendo la causa más importante de letalidad asociada a estas especies. Otras complicaciones incluyen lesión renal aguda, shock, mionecrosis, síndrome compartimental e infecciones secundarias ^9^. El ictus hemorrágico es una complicación que puede ser muy frecuente en pacientes con factores de riesgo preexistentes, como diabetes, nefropatías y enfermedades cardiovasculares ^10^. La actividad coagulante es el resultado de componentes del veneno con actividad similar a la de la trombina, que hidrolizan directamente el fibrinógeno a fibrina, y la actividad procoagulante, que activa los factores de coagulación II y X, lo que resulta en la formación de trombina endógena ^11^. La actividad proteolítica inducida por el veneno provoca lesiones en la pared vascular que provocan hemorragias. En la actividad hemorrágica, la metaloproteinasa P-I, especialmente la batroxasa, tiene actividad fibrinolítica y trombolítica e induce sangrado a través de la digestión de los componentes de la matriz extracelular ^12^.

El caso pediátrico corresponde a un varón de cinco años que acude al centro de salud por un accidente ofídico por género *Bothrops*; con cinco días de evolución. La mordedura fue en el primer dedo del pie izquierdo. Al presentar manifestaciones neurológicas, se solicitó un estudio tomográfico cerebral sin contraste, donde se evidencia una imagen hiperdensa localizada en la asta occipital del ventrículo lateral izquierdo compatible con hemoventriculo unilateral.

En un reporte de caso en Brasil en 2017, se describió a una mujer de 58 años que presentó un accidente cerebrovascular hemorrágico dos días después de sufrir una mordedura de serpiente del género *Bothrops* en el tercer dedo de la mano derecha. La tomografía cerebral reveló un hematoma nucleocapsular derecho y hemorragia en el sistema ventricular derecho ^13^.

Asimismo, en un reporte en Colombia en 2020, se describió a un varón de 59 años que sufrió una mordedura en la parte distal del pulgar derecho, con 12 horas de evolución. En la tomografía computarizada simple de cráneo, se observó hemorragia cerebral témporoparietal masiva, que comprimía los núcleos basales derechos ^14^.

El hemoventrículo es una complicación rara pero grave de las mordeduras de serpientes en niños. Ocurre cuando la acción del veneno ocasiona una hemorragia en el sistema ventricular, constituido por espacios llenos de líquido cefalorraquídeo. El hemoventrículo puede provocar un aumento de la presión dentro del sistema ventricular, con ello aumento de la presión intracraneal y ocasionar complicaciones neurológicas graves como hidrocefalia y síndromes de herniación cerebral.

Los mecanismos exactos por los cuales el veneno de serpiente causa la hemorragia ventricular no se comprenden bien, pero se cree que está relacionado con los efectos tóxicos del veneno en los vasos sanguíneos y las paredes de los ventrículos. Los niños son particularmente susceptibles al hemoventrículo por mordeduras de serpiente porque su cerebro aún está en desarrollo y puede ser más sensible a los efectos tóxicos del veneno. El reconocimiento precoz y el abordaje terapéutico oportuno del emponzoñamiento ofídico son determinantes para prevenir complicaciones graves como el hemoventrículo. Este manejo integral debe contemplar la administración inmediata del antiveneno específico, junto con medidas de soporte orientadas a la estabilización hemodinámica, la corrección del desequilibrio hidroelectrolítico y el control sintomático del dolor y del proceso inflamatorio ^15^.

Ante la sospecha de hemoventrículo, se requieren estudios de imágenes como tomografías computarizadas para confirmar el diagnóstico y evaluar la extensión del sangrado. El tratamiento puede incluir manejo médico o intervención quirúrgica, según la gravedad del sangrado y la presencia de otros síntomas. Es importante buscar atención médica inmediata ante cualquier sospecha de mordedura de serpiente, especialmente en niños, para minimizar el riesgo de hemoventrículo y otras complicaciones potencialmente mortales.

El Perú tiene una biodiversidad notable y cada especie presenta sus propias peculiaridades. La diversidad dentro del complejo de *Bothrops* es especialmente destacable, lo que implica que muchas especies de serpientes nativas con relevancia médica aún no han sido suficientemente estudiadas ^16^.

Existen pocos estudios o reportes que documenten la epidemiología, fisiopatología y características clínicas de la hemorragia ventricular asociada a accidentes ofídicos. Por ello, es necesario realizar estudios que permitan comprender los mecanismos fisiopatológicos involucrados en las manifestaciones neurológicas y determinar por qué los niños podrían presentar una mayor susceptibilidad. Esta evidencia contribuiría a optimizar el manejo clínico y al desarrollo de guías de práctica clínica orientadas a prevenir complicaciones.

En conclusión, la hemorragia intraventricular es una posible complicación del envenenamiento por serpientes del género *Bothrops*., incluso tras mordeduras por ejemplares juveniles, tanto en adultos como en niños. En los dos casos descritos se identificaron demoras en la administración de una dosis adecuada de antiveneno y prácticas prehospitalarias no recomendadas. En el adulto, la coexistencia de hipertensión arterial no tratada impide atribuir la hemorragia exclusivamente al veneno. Ante la aparición de alteraciones neurológicas después de una mordedura botrópica, se sugiere obtener una neuroimagen urgente, preferentemente una tomografía cerebral sin contraste, y reservar la resonancia magnética para casos seleccionados. Garantizar la disponibilidad de antiveneno en los establecimientos del primer nivel de atención y agilizar el traslado fluvial a centros de mayor capacidad resolutiva en la Amazonía podría reducir el riesgo de estas complicaciones.
